# Increased Arrhythmia Risk in Long COVID: A Systematic Review and Meta‐Analysis

**DOI:** 10.1002/joa3.70278

**Published:** 2026-02-09

**Authors:** Amir Reza Boskabadi, Hoorak Poorzand, Ali Vaezi, Sara Afshar, Mohammad Tayyebi, Negar Morovatdar

**Affiliations:** ^1^ Faculty of Medicine Mashhad University of Medical Science Mashhad Iran; ^2^ Vascular and Endovascular Surgery Research Center Mashhad University of Medical Sciences Mashhad Iran; ^3^ Student Research Committee, School of Medicine Tehran University of Medical Sciences Tehran Iran; ^4^ Department of Cardiovascular Disease, Faculty of Medicine Mashhad University of Medical Science Mashhad Iran; ^5^ Clinical Research Unit, Imam Reza Hospital Mashhad University of Medical Sciences Mashhad Iran

**Keywords:** arrhythmias, COVID‐19, Long COVID, post‐acute COVID‐19 syndrome, systematic review

## Abstract

**Background:**

COVID‐19 infection can cause significant long‐term health problems for patients. While there is no universally accepted definition for long COVID, it is usually identified by persistent symptoms that extend past 4 weeks after the initial SARS‐CoV‐2 infection with no other explanation. The cardiovascular system is one of the most important systems involved in long COVID, and even asymptomatic patients have evidence of cardiovascular injury after COVID‐19. This study aims to determine the long‐term risk of developing cardiac arrhythmias after SARS‐CoV‐2 infection.

**Methods:**

A comprehensive systematic search on Scopus, PubMed, Science Direct, and Web of Science databases was performed on August 24th, 2025. Cohort articles consisting of a healthy control group with no history of COVID‐19 infection and individuals who recovered from COVID‐19 for at least 30 days were included. Hazard Ratio (HR) and 95% confidence intervals (CI) were estimated using random‐effect models.

**Results:**

Fourteen studies were eligible for the meta‐analysis. The overall arrhythmia risk was higher in patients with long COVID (HR: 1.74, 95% CI [1.39, 2.10], *I* ^2^ = 99.65%). Specific arrhythmias examined included atrial fibrillation (HR: 1.49, 95% CI [1.24, 1.73], *I* ^2^ = 98.57%), sinus tachycardia (HR: 1.69, 95% CI [1.21, 2.18], *I* ^2^ = 99.51%), sinus bradycardia (HR: 1.58, 95% CI [1.50, 1.66], *I* ^2^ = 65.80%), and ventricular arrhythmias (HR: 1.72, 95% CI [1.48, 1.95], *I* ^2^ = 96.89%). Patients with a more severe initial infection were at a higher risk of developing arrhythmias.

**Conclusions:**

The risk of developing cardiac arrhythmias is increased after COVID‐19 infection in the long term.

## Graphical abstract

This meta‐analysis included 14 studies. Long COVID was associated with a higher risk of atrial fibrillation, sinus tachycardia, sinus bradycardia, and ventricular arrhythmias. Patients with more severe COVID‐19 infection were at a higher risk of developing arrhythmias in the long term.

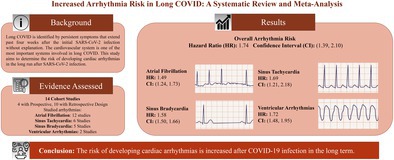

## 1 Introduction

COVID‐19 is an infection caused by the SARS‐CoV‐2 virus that primarily affects the respiratory system and thus can manifest as viral pneumonia and present with fever, malaise, shortness of breath, and cough. However, this virus can involve different organ systems such as the musculoskeletal, gastrointestinal, endocrine, and renal systems, causing different manifestations [1]. The cardiovascular system is particularly susceptible to complications arising from COVID‐19 infection, making it an area of concern. These complications include myocarditis, cardiac arrhythmias, myocardial infarction, heart failure, and thromboembolic events [2, 3, 4, 5]. They can contribute to the morbidity and mortality of the disease [6]. Several mechanisms have been proposed for cardiovascular injury in COVID‐19 disease. SARS‐CoV‐2 gains entry into the cells through angiotensin‐converting enzyme 2 (ACE2) and can disrupt the normal signaling pathways mediated by ACE2 in both heart and endothelial cells, thus causing myocardial injury [7, 8]. Also, infection with SARS‐CoV‐2 can be a trigger for systemic inflammation and cytokine release storm, which can lead to cardiac injury and cardiomyopathy, plaque destabilization, and coagulopathy [3, 7, 8].

The phrase “Long COVID” was first introduced by Elisa Perego, a COVID‐19 survivor, to describe lingering symptoms after acute infection, and later the term “long haulers” was employed by Watson and Yong [9]. Although there is currently no universally accepted definition, the National Health Service of the UK defines long COVID as the “presence of lingering symptoms lasting beyond four weeks beyond the initial SARS‐CoV‐2 infection without other explanation”, divided into two phases: ongoing symptomatic phase (4 to 12 weeks) and post‐COVID‐19 syndrome (PCS) (more than 12 weeks), based on symptom duration. It is not a single condition, but rather a set of overlapping entities with different etiologies, risk factors, and outcomes. Additionally, other terms have been used to describe long COVID, including post‐acute sequelae of COVID‐19 syndrome (PASC), post‐acute COVID‐19, and long‐haul COVID‐19 [10, 11].

Individuals with long COVID often report a variety of symptoms, including fatigue, arthralgia, myalgia, gustatory and olfactory dysfunction, dizziness, insomnia, memory loss, difficulty concentrating, headaches, palpitations, dyspnea, and chest pain [11, 12, 13]. While the exact pathophysiology underlying long COVID is not yet fully elucidated, researchers have proposed several mechanisms, including oxidative stress, immunologic abnormalities, inflammatory damage, and viral‐specific variations [14]. Emerging evidence suggests that SARS‐CoV‐2 infections can have a lasting impact on various organ systems, including fibrosis‐like changes and ground‐glass opacities in the lungs [15], an increased risk of new‐onset diabetes [15], renal function impairment [16], peripheral neuropathy, and myopathy [17, 18].

The cardiovascular system is one of the most important systems involved in long COVID. Cardiovascular symptoms including chest pain, palpitations, and dyspnea are prevalent among patients. Even asymptomatic patients show evidence of cardiovascular injury, such as myocardial inflammation, systolic and diastolic dysfunction, and ischemic changes in cardiac MRI and echocardiography [10]. The American College of Cardiology classifies PASC into two distinct groups based on the absence or presence of objectively confirmed cardiovascular disease. The term PASC‐cardiovascular disease (PASC‐CVD) is used to describe patients with a discernible myocardial, pericardial, arrhythmic, or vascular condition that appears 4 weeks after the initial SARS‐CoV‐2 infection. On the other hand, the term PASC‐cardiovascular syndrome (PASC‐CVS) is characterized by the persistence of cardio‐pulmonary syndromes in the absence of cardiovascular disease [19]. This study aims to investigate the risk of cardiac arrhythmia development as a long‐term consequence of COVID‐19.

## 2 Method

### 2.1 Study Protocol

We conducted this meta‐analysis according to the latest version of Preferred Reporting Items for Systematic Reviews and Meta‐analyses (PRISMA) guidelines and checklists [20]. This study was also registered and approved by the research board and the medical ethics committee at Mashhad University of Medical Sciences (code of ethics: IR.MUMS.IRH.REC.1402.014). Also, the study protocol was registered in PROSPERO (ID: CRD42024587028).

### 2.2 Search Strategy

Two independent authors (A.R.B. and A.V.) conducted a systematic literature search in four scientific databases (Scopus, PubMed, Science Direct, and Web of Science) from inception to August 24th, 2025. We used keywords combined with Medical Subject Headings (MeSH) terms such as “arrhythmia,” “tachycardia,” “Post‐Acute COVID‐19 Syndromes,” “Long Haul COVID‐19,” and “Post‐Acute Sequelae of SARS‐CoV‐2 Infection” in combination with Boolean operators in the advanced search engine. No language or country restrictions were applied (any non‐English record was translated). The search strategy details are provided in the Table S1.

### 2.3 Study Selection and Eligibility Criteria

For this review, Long COVID was defined in line with the included studies as patients who had documented positive results for COVID‐19 diagnostics and had recovered from the acute COVID‐19 for at least 30 days. The inclusion criteria consisted of observational cohort studies that enrolled long COVID patients, without documented cardiac disease before COVID‐19 infection, with controls that had a negative COVID‐19 diagnostic test and/or no history of COVID‐19 infection. Studies without a control group, reviews, case reports, case series, conference abstracts, and editorials were excluded.

The same two authors (A.R.B. and A.V.) investigated the retrieved reports for relevant articles. After manually removing duplicate records, the title and abstract of the remaining references were screened. In the next step, we reviewed the full texts of the documents to assess their eligibility for inclusion. To increase the power of the search, we also searched the references of the included papers to obtain any missed reports. Finally, the two authors (A.R.B. and A.V.) compared their results, and disagreements were addressed through discussion or consultation with a third author (N.M.).

### 2.4 Data Extraction and Quality Assessment

Two independent authors (A.R.B. and A.V.) conducted the data extraction based on the following format: First author's name, publication year, region, study period, type of the cohort—follow‐up (the mean time each patient was under observation), number of patients and controls, days past COVID diagnosis, and type of arrhythmias. The Joanna Briggs Institute (JBI) checklist for cohort studies [21] was utilized for critical appraisal. The same two authors carried out the quality assessment and a third author (N.M.) was consulted in case of discrepancy in results, both in data extraction and critical appraisal sections.

### 2.5 Statistical Analysis

The pooled effect estimates are shown as pooled hazard ratios (HRs) with 95% confidence intervals (CIs) and corresponding *p*‐values. The *I* ^2^ statistic was utilized to measure heterogeneity among the studies. The value of more than 50% was regarded as moderate heterogeneity. The selection of a fixed‐effect or a random‐effect model was determined by the degree of heterogeneity among the studies. The potential influence of publication bias was investigated by generating funnel plots. Sensitivity analysis was conducted to assess the robustness of the pooled estimates. Statistical analysis was carried out using STATA version 17 (College State, TX, USA).

## 3 Results

### 3.1 Literature Search

**FIGURE 1:**
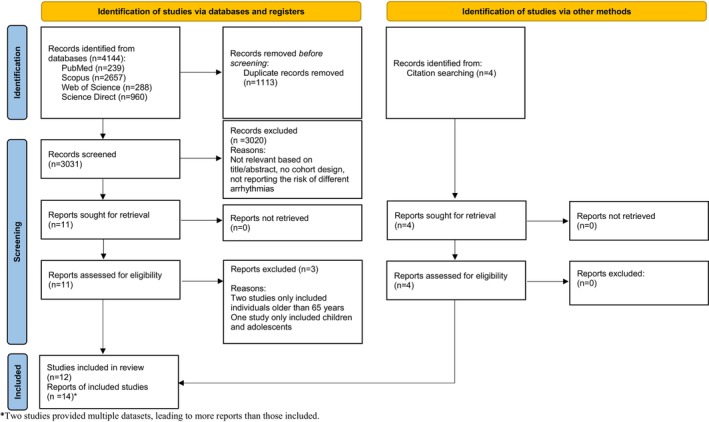
The PRISMA flow diagram of the systematic review.

In total, the primary literature search was conducted on August 24th, 2025 in four databases and yielded 4144 records, including preprints. After removing 1113 duplicates, two independent authors (A.R.B. and A.V.) performed the first screening stage by reviewing the title and abstract of the 3031 results. Papers without a healthy control group and cohort design and papers without reporting the arrhythmia risk were excluded. In the next step, the full‐text copies of the remaining 17 reports were acquired and carefully read to select articles meeting our inclusion criteria. Four additional studies were found through citation searching and were added to the list. Two of the articles (by Lam et al. [22] and Ojeda‐Fernández et al. [23]) provided two different datasets and therefore, each dataset was counted as a separate effect size. Finally, 14 studies were included in this systematic review. The PRISMA diagram of the review is shown in Figure 1.

### 3.2 Studies' Characteristics

Fourteen included studies were published between 2022 and 2024, and originated in the USA [24, 25, 26, 27], UK [22, 28, 29], Hong Kong [22], Singapore [30, 31], Spain [32, 33], and Italy [23, 32]. Four studies [27, 28, 29, 33] had a prospective design while the other 10 [22, 23, 24, 25, 26, 30, 31, 32] were retrospective cohorts. The mean follow‐up period among the studies varied from 84 days to 20 months. The number of patients and controls ranged from 3578 to 3 849 967 and 849 to 8 980 919, respectively. Male to female ratio and mean age ranged widely from 9.3 to 0.71 and 35 to 68 years for both the patient and control groups. Studies defined their control groups mainly as having no COVID‐19 symptoms and/or negative PCR tests. The time of COVID‐19 diagnosis was at least 30 days before enrollment. All studies defined arrhythmias according to the International Classification of Diseases (ICD) [34]. All studies accounted for key demographic and cardiovascular risk factors (e.g., age, hypertension, smoking, etc.) through adjustment methods such as propensity score matching, inverse probability matching, or multivariable analysis. The study characteristics are summarized in Table 1.

**TABLE 1:** Characteristics of the studies exploring the risk of arrhythmias in long COVID patients.

First author (year of publication)	Study period	Country	Type of study	Follow‐up	Number of patients (% female)	Mean age patients	Control group	Number of controls (% female)	Mean age controls
Xie Y. (2022) [1]	March 2020–January 2021	USA	Prospective cohort	347 days	153 760 (11%)	61.42 ± 15.64	Contemporary control: no evidence of SARS‐COV infection	5 637 647 (9.7%)	63.46 ± 16.23
Wang W. (2022) [2]	January 2019–March 2022	USA	Retrospective cohort	1 year	690 892 (56.9%)	43.2 ± 16.2	People with no symptoms and a negative COVID‐19 test (Vaccinated people were excluded)	690 892 (56.9%)	43.1 ± 16.1
Ortega‐Paz L. (2022) [3]	February 2020–December 2020	Spain and Italy	Retrospective cohort	1 year	3578 (43.3%)	63.1 ± 17.3	Patients with no symptoms and a negative PCR test	849 (58.4%)	48.8 ± 19.1
Mabila S. (2023) [4]	March 2020–November 2021	USA	Retrospective cohort	20 months	122 424 (19%)	Not Reported	Negative PCR test	875 361 (20%)	Not Reported
Wan E. (2022) [5]	March 2020–November 2020	UK	Prospective cohort	18 months	7584 (50.4%)	66.1 ± 8.6	Patients without a COVID‐19 diagnosis for the whole duration of the study	75 790 (50.9%)	66.3 ± 8.3
Lim J. (2024) [6]	September 2021–November 2021	Singapore	Retrospective cohort	300 days	106 012 (44.2%)	51 ± 17.25	Patients with negative PCR tests and no reports of COVID‐19 infection within 300 days	1 684 085 (51.9%)	48 ± 17.7
Tintore C. (2024) [7]	March 2020–May 2020	Spain	Prospective cohort	317 days	33 674 (59.3%)	66	Patients without a positive Covid test match the patients' group	130 672 (59.7%)	66
Lam I. (2024) [8]	April 2020–May 2022	Hong Kong	Retrospective cohort	146 days	3 849 967 (56%)	54.1 ± 17.4	Patients without a positive Covid test match the patients' group	3 850 839 (55.9%)	54.2 ± 18.2
Lam I. (2024) [8]	March 2020–May 2021	UK	Retrospective cohort	243 days	183 091 (55%)	68.1 ± 8.5	Patients without a positive Covid test match the patients' group	409 176 (55.3%)	68.1 ± 8.1
Ojeda‐Fernandez L. (2023) [9]	February 2020–June 2020	Italy	Retrospective cohort	222 days	59 545 (53.8%)	64.97 ± 14.97	Patients without a COVID‐19 diagnosis during the follow‐up	196 091 (49.95%)	66.65 ± 14.73
Ojeda‐Fernandez L. (2023) [9]	October 2020–May 2021	Italy	Retrospective cohort	237 days	425 600 (52.6%)	59.26 ± 13.48	Patients without a COVID‐19 diagnosis during the follow‐up	1 316 933 (51.78%)	60.12 ± 13.87
Wee L. (2024) [10]	December 2021—March 2022	Singapore	Retrospective cohort	300 days	375 903 (50.92%)	48 ± 17.66	Patients with a negative test from the same population and time period	619 379 (52.55%)	47 ± 13.35
Daugherty S. (2021) [11]	January 2020—October 2020	USA	Retrospective cohort	87 days	266 586 (52.4%)	41.7 ± 13.9	A comparator group with no clinical diagnosis related to COVID‐19	8 980 919 (49.7%)	42.4 ± 13.6
Rezel‐Potts E. (2022) [12]	February 2021—January 2022	UK	Prospective cohort	12 weeks	428 650 (55.58%)	35 (22–50)	A matched control group from the same population with no history of COVID‐19	428 650 (55.58%)	35 (22–50)

Regarding the critical appraisal, using the JBI checklist [21] to assess the quality of the selected studies, one study received a score of 10, and the others were given the maximum score of 11 (Table S2).

### 3.3 Overall Arrhythmia Risk

**FIGURE 2:**
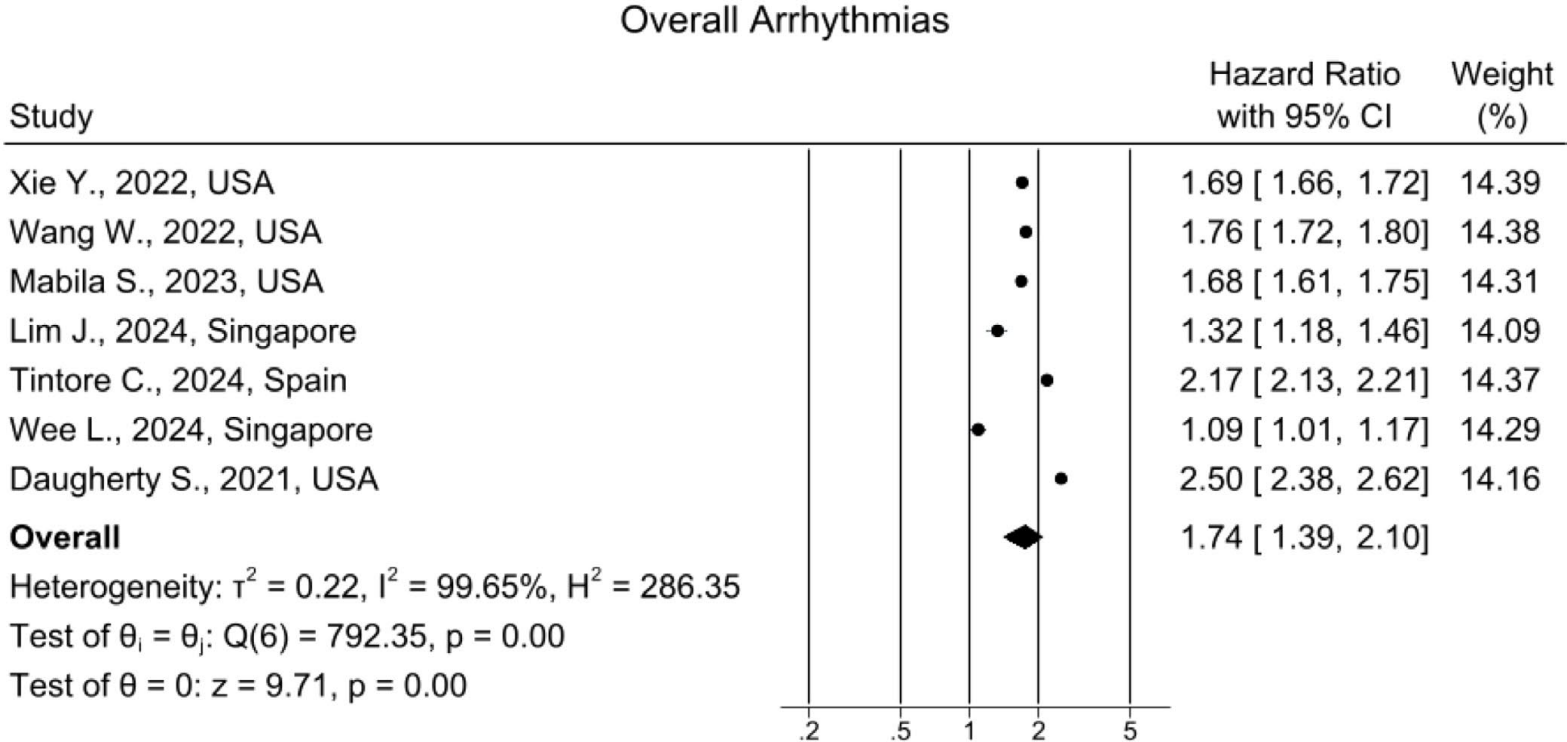
Forest plot for the risk of overall arrhythmias in long COVID.

Seven studies evaluated the overall risk of developing arrhythmias. (Figure 2). Patients with long COVID had a 74% higher risk of developing arrhythmias compared to control groups as determined by a random‐effects model (HR: 1.74, 95% CI [1.39, 2.10]; *I* ^2^ = 99.65%). The funnel plot demonstrated some asymmetry (Figure S1). Results remained similar after sensitivity analysis (Figure S2).

### 3.4 Atrial Fibrillation

**FIGURE 3:**
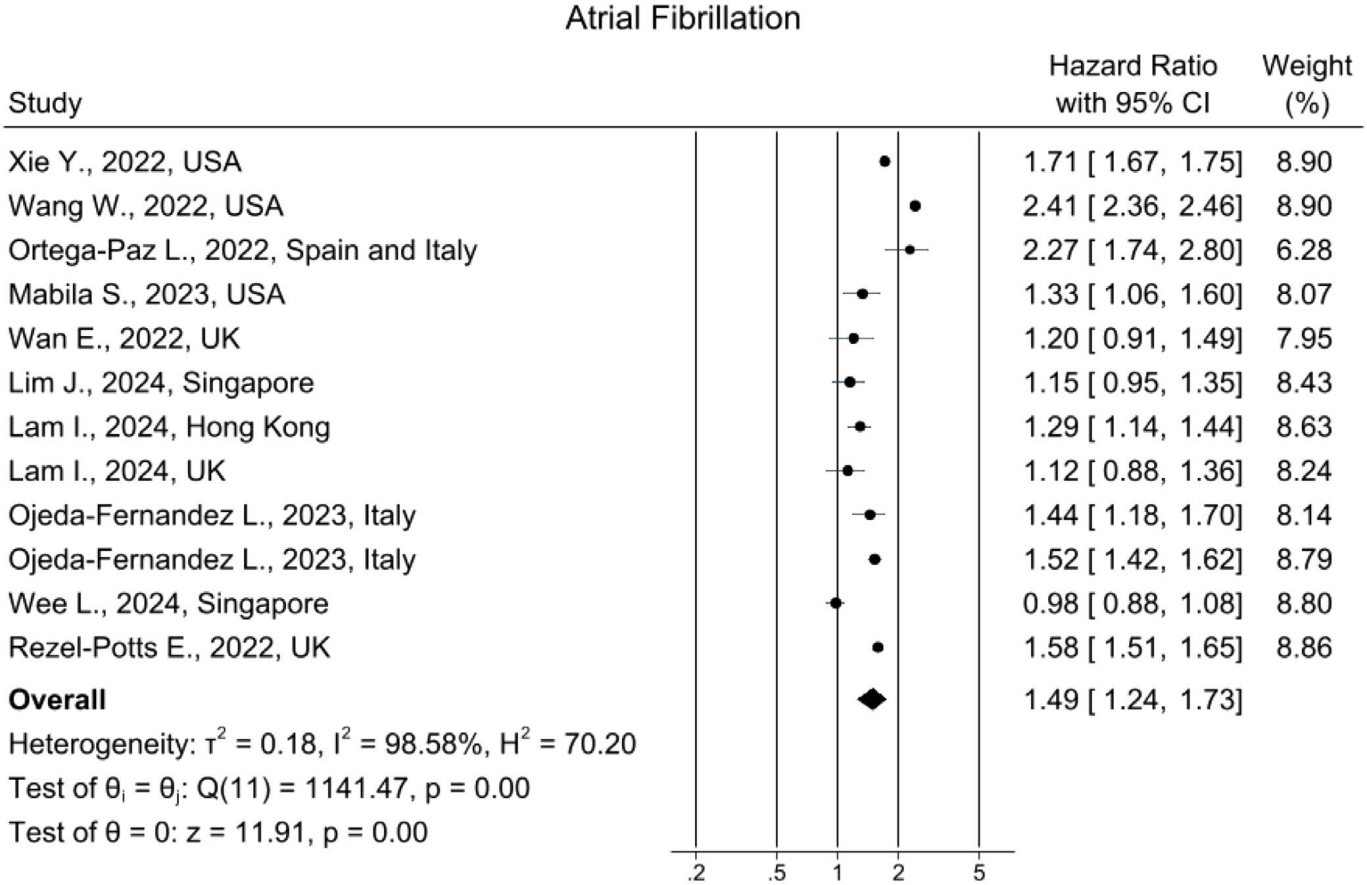
Forest plot for the risk of atrial fibrillation in long COVID.

Twelve studies calculated the incidence of atrial fibrillation (AF). AF risk increased by 49% in long COVID patients compared to controls (HR: 1.49, 95% CI [1.24, 1.73], *I* ^2^ = 98.57%), using a random‐effects model (Figure 3). Although the funnel plot showed mild asymmetry (Figure S3), both Egger's and Beggs' tests indicated no statistically significant publication bias (*p =* 0.95 and 0.24, respectively). No substantial changes were observed when sensitivity analysis was conducted (Figure S4).

### 3.5 Sinus Tachycardia

**FIGURE 4:**
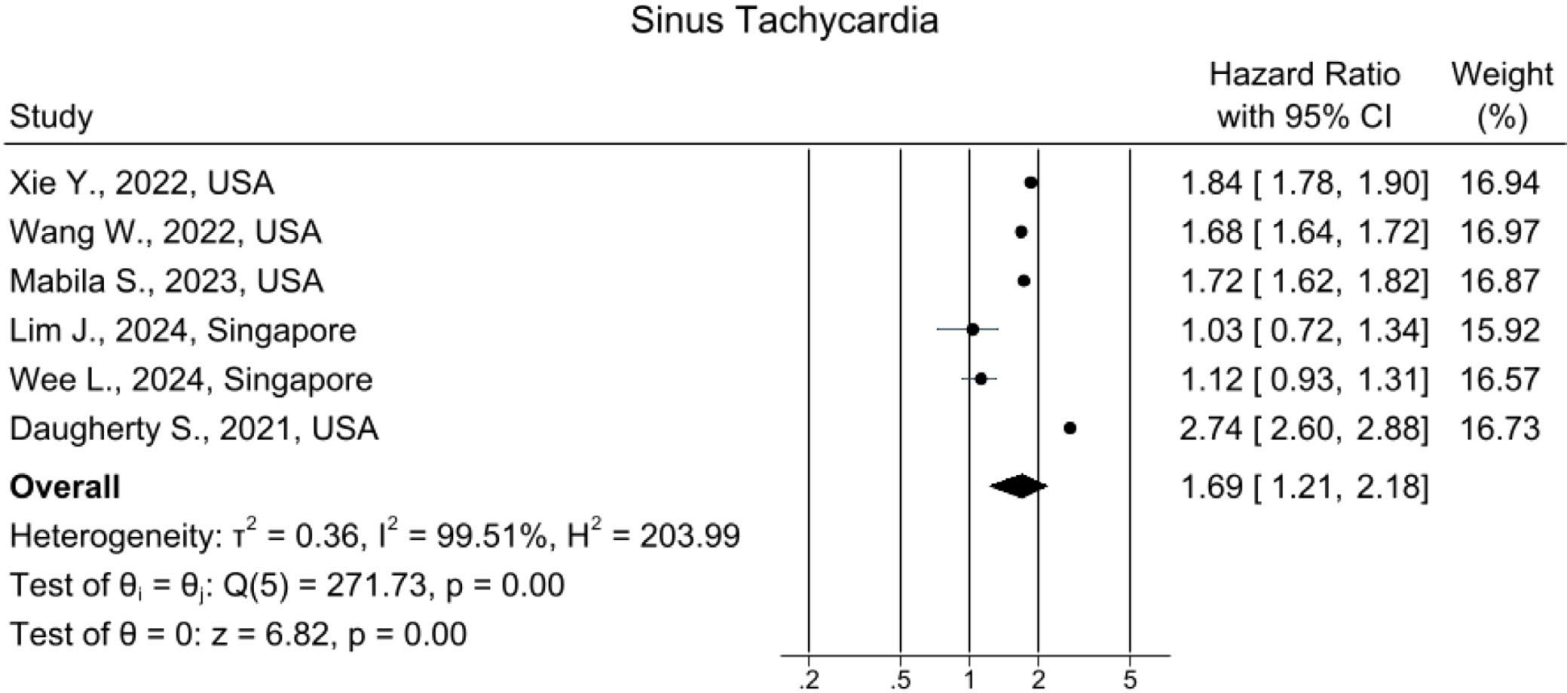
Forest plot for the risk of sinus tachycardia in long COVID.

Six articles calculated the risk of developing sinus tachycardia after COVID‐19 infection in the long term. The pooled HR was 1.69, 95% CI [1.21, 2.18] (*I* ^2^ = 99.51%), which showed a meaningful difference between the COVID‐19 and the healthy group (Figure 4). The funnel plot showed some asymmetry (Figure S5). Sensitivity analysis yielded results consistent with the main analysis (Figure S6).

### 3.6 Sinus Bradycardia

**FIGURE 5:**
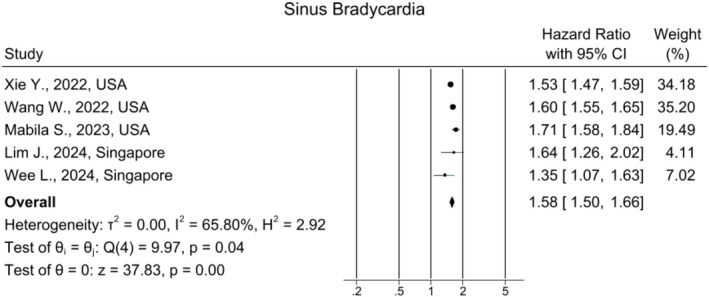
Forest plot for the risk of sinus bradycardia in long COVID.

Five studies reported the burden of sinus bradycardia in the long COVID group. Our analysis showed an HR of 1.58, 95% CI:[1.50, 1.66] (*I* ^2^ = 65.80%) using a random‐effects model, which demonstrates a notable increase (Figure 5). The funnel plot did not show any significant publication bias (Figure S7). The sensitivity analysis confirmed the robustness of the findings, with minimal variations in effect sizes (Figure S8).

### 3.7 Ventricular Arrhythmias

**FIGURE 6:**
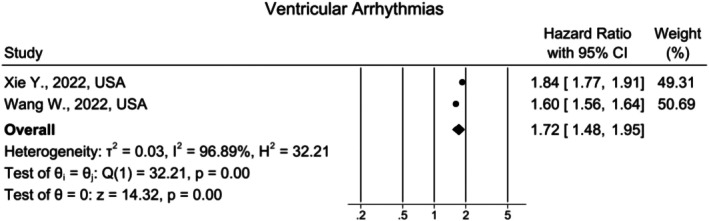
Forest plot for the risk of ventricular arrhythmias in long COVID.

Only two studies reported the burden of ventricular arrhythmias between the long COVID and the control group. Both articles showed that the incidence of ventricular arrhythmias was significantly increased in the long COVID group. Using the random‐effects model, the overall HR was 1.72, 95% CI [1.48, 1.95] (*I* ^2^ = 96.89%) (Figure 6).

### 3.8 The Severity of the Initial COVID‐19 Infection and the Risk of Arrhythmia

The studies by Lim et al. [30], Wee et al. [31] and Xie et al. [27] divided the patients into three groups based on the severity of the initial infection: outpatient (mild), inpatient, and intensive care unit (ICU). In the study by Lim et al. [30], the overall arrhythmia risk for mild cases was not significantly different from the control group (HR: 1.11, 95% CI [0.94, 1.30]). However, both inpatient and ICU groups had a significantly higher risk of developing arrhythmias (HR: 2.95, 95% CI [1.80, 4.85] for inpatient, and HR: 4.65, 95% CI [2.53, 8.55]). The research by Wee et al. [31] also found similar results (HR: 0.99, 95% CI [0.91, 1.09] for the mild group, HR: 3.30, 95% CI [2.30, 4.74] for the inpatient group, and HR: 4.54, 95% CI [2.65, 7.77] for the ICU group). Meanwhile, in the Xie et al. study [27], all three patient groups were at higher risk of cardiac arrhythmias in the long term (HR: 1.33, 95% CI [1.29–1.38] for the outpatient group, HR: 3.89, 95% CI [3.55–4.27] for the inpatient group, and HR: 7.93, 95% CI [7.00–8.98] for the ICU group). Wang et al. [26], divided the patients into two groups (inpatient and outpatient) and exhibited an increased risk for all of the studied arrhythmias (AF, tachycardia, bradycardia, ventricular arrhythmias) in the inpatient group, while the outpatient group only showed an increased risk for tachycardia and bradycardia. The reviewed studies suggest a relationship between the severity of COVID‐19 and arrhythmia risk. The ICU patients exhibited a higher risk of arrhythmia compared to outpatients and inpatient groups, with varying HRs reported across studies. Since studies used different adjustment methods for potential confounders, the results are not directly comparable due to variability in methodologies, and thus, a meta‐analysis was not performed.

## 4 Discussion

Our meta‐analysis shows that patients with post‐acute COVID‐19 have a higher risk of AF, sinus tachycardia, sinus bradycardia, and ventricular arrhythmias compared to controls. The studies showed high heterogeneity, which may be due to differences in patient populations, follow‐up lengths, study designs (retrospective vs. prospective), surveillance approaches (continuous versus episodic electrocardiographic recording), and geographical differences, including the predominance of viral variants across regions. These results highlight the long‐term cardiovascular effects of SARS‐CoV‐2 infection.

The most observed arrhythmia in the acute SARS‐CoV‐2 cases is AF. Individuals with advanced age, critical illness, male sex, uncontrolled hypertension, and diabetes are more prone to developing AF [35]. The presence of AF in patients experiencing acute COVID‐19 infection was linked to a higher chance of all‐cause mortality [35], but its relevance in post‐COVID is not well investigated. Our meta‐analysis showed a significant increase in AF occurrence in post‐acute COVID‐19 patients in comparison to the control groups. A previous meta‐analysis by Zuin et al. [36], which included five studies, also showed that post‐acute COVID patients are at a higher risk of developing AF. A nested case–control study by Rosh et al. [37], demonstrated that a previous SARS‐CoV‐2 infection can predispose patients to new‐onset AF. The association was meaningful in the lag‐time analysis, but this connection was weakened as the time interval increased and stabilized around a lag‐time of 20 days. A cohort study by Wang et al. [26], demonstrated that patients with a previous COVID‐19 infection were more likely to experience atrial flutter and fibrillation (HR: 2.407, 95% CI [2.296, 2.523]) relative to the healthy control group. These findings emphasize the importance of evaluating patients with long COVID and identifying high‐risk patients for developing AF.

Sinus tachycardia is frequently observed in post‐acute COVID‐19 patients and can be responsible for some of the symptoms they experience. In a study by Aranyo et al. [38], patients with post‐COVID syndrome who had sinus tachycardia had more common complaints of palpitations, dyspnea, headache, and dizziness. Another article by Llach et al. [39] showed similar results in patients with inappropriate sinus tachycardia. These results align with our study, highlighting the frequent occurrence of tachycardia in post‐acute COVID‐19 patients.

Bradycardia can also be a manifestation of acute COVID‐19 infection. It is a common occurrence in COVID‐19 cases, but its significance is controversial. Some studies found that bradycardia was associated with higher ICU admissions, hospital stays, and mortality [40], while in others, there was no correlation between the severity of bradycardia and poor outcomes [41]. In our study, post‐acute COVID‐19 patients had an increased risk of developing sinus bradycardia in the long term as well. In a study by Zhou et al. [42], the incidence of bradycardia in post‐acute COVID‐19 patients was 29.9%, with 7.2% of COVID survivors having significant sinus bradycardia (heart rate < 50). The bradycardia appeared to be self‐limiting. In another study by Afroze et al. [43], 5% of outpatient and 2% of inpatient COVID survivors showed sinus bradycardia on their ECG after one‐month follow‐up, which was significantly less than sinus tachycardia (28% outpatient and 11% inpatient). The moderate heterogeneity observed in our sinus bradycardia analysis may reflect differences across studies in patient population, follow‐up duration, methods of outcome ascertainment, variations in age distribution, and baseline cardiovascular risk. Further studies are warranted to clarify the clinical relevance of sinus bradycardia and its association with symptoms and long‐term outcomes in COVID‐19 survivors.

Interestingly, both sinus tachycardia and sinus bradycardia were observed in post‐COVID patients. This apparent paradox can be explained by autonomic dysfunction, where some individuals develop postural orthostatic tachycardia syndrome (POTS)‐like syndromes, while others manifest vagally‐mediated bradyarrhythmias. Inflammatory or fibrotic injury to the sinus node may further contribute to these divergent phenotypes. Such differences likely reflect heterogeneity in patient age, comorbidities, and underlying cardiac autonomic balance [44]. Several other studies have also mentioned autonomic dysfunction as a potential mechanism responsible for arrhythmias after COVID‐19. A systematic review by Hyo‐Weon Suh et al. [45], showed that patients with long COVID had changes in heart rate variability (HRV) parameters, including a reduction in the standard deviation of normal‐to‐normal RR intervals (SDNN), which indicates parasympathetic inhibition.

Ventricular arrhythmias are common among patients who are hospitalized with acute COVID‐19. According to a meta‐analysis by Tan et al. [46], the incidence of ventricular arrhythmias in hospitalized patients was 5%, with 95% CI [4%, 6%]. It was significantly associated with mortality and sudden cardiac death. Unlike acute COVID, there is not much evidence on the post‐acute COVID patients. In our meta‐analysis, we found that patients with a history of previous COVID‐19 infection were more likely to experience ventricular arrhythmias. In a study by Ingul et al. [47], which performed Holter monitoring on 200 patients with long COVID, 18% of patients had a significant number of premature ventricular contractions (PVCs) (more than 200 beats per 24 h), and 5% had non‐sustained ventricular tachycardias, but the study lacked a control group for arrhythmias. There have also been several case reports regarding the development of ventricular arrhythmias in post‐COVID patients. A study by Rosca et al. [48] described three young males without prior cardiovascular disease who experienced non‐sustained ventricular tachycardia (NSVT) approximately 4 weeks after recovering from COVID‐19. A similar case report was published by El Hajjar et al. [49] describing a young patient who developed ventricular tachycardia after the resolution of COVID‐19. Several mechanisms can contribute to the development of ventricular arrhythmias in post‐COVID patients. A paper by Hamdy et al. [50] reported that post‐COVID patients with ventricular arrhythmias had significantly lower functional status and showed subclinical myocardial damage in echocardiography. In addition, patients with ventricular arrhythmias had higher levels of inflammatory biomarkers. More research is needed on ventricular arrhythmias since they are associated with mortality and adverse outcomes.

The severity of the COVID‐19 infection is a key factor contributing to the long‐term consequences. Several studies have shown that even patients with absent to mild symptoms can suffer from long COVID symptoms like cough, dyspnea, headaches, anosmia, and ageusia [51, 52, 53]. However, patients with more severe COVID‐19 disease are more likely to develop PASC in the future [54]. In a paper by Wiemken et al. [55], patients who were hospitalized and patients requiring intensive care unit (ICU) were more likely to experience cardiovascular events in the long run. ICU‐admitted patients exhibited a heightened risk for cardiac arrhythmias compared with non‐ICU hospitalized patients and non‐hospitalized patients (HR: 1.22, 95% CI [1.17–1.27] for ICU vs. hospital‐admitted patients, HR: 1.75, 95% CI [1.65–1.87] for ICU vs. non‐hospitalized patients). In our systematic review, three articles confirm these findings by showing an increased risk of cardiac arrhythmias in hospital‐admitted and ICU patients. These studies indicate that the intensity of the initial COVID‐19 infection directly impacts the risk of PASC‐CVD, including arrhythmias. However, it is essential to note that ICU patients often have more severe comorbidities and receive distinct treatments. These factors may introduce confounding, which could affect the interpretation of the results. While the included studies employed multivariable adjustment, the heterogeneity in study methodologies and the presence of confounding factors limit the ability to directly compare or combine the results.

## 5 Strengths and Limitations

To our knowledge, this is the first systematic review and meta‐analysis on the incidence of different cardiac arrhythmias in post‐acute COVID‐19 patients. Also, this meta‐analysis included studies of high methodological quality and a large number of patients. The included studies encompassed a large number of patients, which increased the precision of the estimates.

The main limitation of this meta‐analysis is the limited number of studies for some of the analyses. In addition, we could not perform a meta‐analysis to assess the relationship between the risk of arrhythmias and initial COVID severity, due to the limited number of studies and different methodologies used to evaluate these relationships.

## 6 Conclusion

COVID‐19 infection can potentially increase the risk of developing different cardiac arrhythmias in the long term. Necessary measures need to be taken to manage patients with long COVID, thereby increasing the quality of life and preventing adverse health events. Early diagnosis may help prevent adverse outcomes in patients.

## Author Contributions

N.M. developed the original idea. A.R.B. and A.V. conducted the literature search and title/abstract screening, consulting N.M. in cases of discrepancy. S.A. and M.T. explored additional sources to find relevant articles and contributed to the study planning. A.R.B., A.V., N.M., and H.P. reviewed the full texts of relevant papers. A.R.B., A.V., and H.P. handled data extraction and quality assessment of the studies. N.M. and A.R.B. performed the meta‐analysis. A.R.B. and A.V. drafted the initial draft. S.A. and M.T. reviewed and improved the draft. All authors read and approved the final version of the manuscript.

## Funding

This study was funded by the Mashhad University of Medical Sciences.

## Disclosure

No human participant was involved in this study. The study protocol was registered in PROSPERO (ID: CRD42024587028).

## Conflicts of Interest

The authors declare no conflicts of interest.

## Supporting information


**Data S1:** joa370278‐sup‐0001‐supinfo.docx.

## Data Availability

The data that supports the findings of this study are available in the Supporting Information of this article.

## References

[1] O. P. Mehta , P. Bhandari , A. Raut , S. E. O. Kacimi , and N. T. Huy , “Coronavirus Disease (COVID‐19): Comprehensive Review of Clinical Presentation,” Frontiers in Public Health 8 (2021): 582932. 33520910 10.3389/fpubh.2020.582932 PMC7844320

[2] F. Farshidfar , N. Koleini , and H. Ardehali , “Cardiovascular Complications of COVID‐19,” JCI Insight 6, no. 13 (2021): 1–15. 10.1172/jci.insight.148980 PMC8410051 34061779

[3] T. Iba , J. H. Levy , J. M. Connors , T. E. Warkentin , J. Thachil , and M. Levi , “The Unique Characteristics of COVID‐19 Coagulopathy,” Critical Care 24, no. 1 (2020): 360. 32552865 10.1186/s13054-020-03077-0 PMC7301352

[4] S. K. Kunutsor and J. A. Laukkanen , “Cardiovascular Complications in COVID‐19: A Systematic Review and Meta‐Analysis,” Journal of Infection 81, no. 2 (2020): e139–e141. 32504747 10.1016/j.jinf.2020.05.068 PMC7832225

[5] B. Long , W. J. Brady , A. Koyfman , and M. Gottlieb , “Cardiovascular Complications in COVID‐19,” American Journal of Emergency Medicine 38, no. 7 (2020): 1504–1507. 32317203 10.1016/j.ajem.2020.04.048 PMC7165109

[6] D. Bandyopadhyay , T. Akhtar , A. Hajra , et al., “COVID‐19 Pandemic: Cardiovascular Complications and Future Implications,” American Journal of Cardiovascular Drugs 20 (2020): 311–324. 32578167 10.1007/s40256-020-00420-2 PMC7310596

[7] V. Petrovic , D. Radenkovic , G. Radenkovic , V. Djordjevic , and M. Banach , “Pathophysiology of Cardiovascular Complications in COVID‐19,” Frontiers in Physiology 11 (2020): 575600. 33162899 10.3389/fphys.2020.575600 PMC7583694

[8] Y.‐H. Zhao , L. Zhao , X.‐C. Yang , and P. Wang , “Cardiovascular Complications of SARS‐CoV‐2 Infection (COVID‐19): A Systematic Review and Meta‐Analysis,” Reviews in Cardiovascular Medicine 22, no. 1 (2021): 159–165. 33792257 10.31083/j.rcm.2021.01.238

[9] E. Perego , F. Callard , L. Stras , B. Melville‐Johannesson , R. Pope , and N. Alwan , “Why We Need to Keep Using the Patient Made Term ‘Long Covid’,” (2020).

[10] B. Raman , D. A. Bluemke , T. F. Lüscher , and S. Neubauer , “Long COVID: Post‐Acute Sequelae of COVID‐19 With a Cardiovascular Focus,” European Heart Journal 43, no. 11 (2022): 1157–1172. 35176758 10.1093/eurheartj/ehac031 PMC8903393

[11] M. Debski , V. Tsampasian , S. Haney , et al., “Post‐COVID‐19 Syndrome Risk Factors and Further Use of Health Services in East England,” PLOS Global Public Health 2, no. 11 (2022): e0001188. 36962824 10.1371/journal.pgph.0001188 PMC10022108

[12] Q. Han , B. Zheng , L. Daines , and A. Sheikh , “Long‐Term Sequelae of COVID‐19: A Systematic Review and Meta‐Analysis of One‐Year Follow‐Up Studies on Post‐COVID Symptoms,” Pathogens 11, no. 2 (2022): 269. 35215212 10.3390/pathogens11020269 PMC8875269

[13] A. D. Proal and M. B. VanElzakker , “Long COVID or Post‐Acute Sequelae of COVID‐19 (PASC): An Overview of Biological Factors That May Contribute to Persistent Symptoms,” Frontiers in Microbiology 12 (2021): 1494. 10.3389/fmicb.2021.698169 PMC8260991 34248921

[14] J. D. Pierce , Q. Shen , S. A. Cintron , and J. B. Hiebert , “Post‐COVID‐19 Syndrome,” Nursing Research 71, no. 2 (2022): 164–174. 34653099 10.1097/NNR.0000000000000565

[15] C. Bellia , A. Andreadi , I. D'Ippolito , et al., “Prevalence and Risk of New‐Onset Diabetes Mellitus After COVID‐19: A Systematic Review and Meta‐Analysis,” Frontiers in Endocrinology 14 (2023): 1215879. 37732118 10.3389/fendo.2023.1215879 PMC10507325

[16] V. Rai , “COVID‐19 and Kidney: The Importance of Follow‐Up and Long‐Term Screening,” Life 13, no. 11 (2023): 2137. 38004277 10.3390/life13112137 PMC10672056

[17] A. L. Oaklander , A. J. Mills , M. Kelley , et al., “Peripheral Neuropathy Evaluations of Patients With Prolonged Long COVID,” Neurology Neuroimmunology & Neuroinflammation 9, no. 3 (2022): e1146. 35232750 10.1212/NXI.0000000000001146 PMC8889896

[18] D. S. Saif , R. A. Ibrahem , and M. A. Eltabl , “Prevalence of Peripheral Neuropathy and Myopathy in Patients Post‐COVID‐19 Infection,” International Journal of Rheumatic Diseases 25, no. 11 (2022): 1246–1253. 35915515 10.1111/1756-185X.14409 PMC9538868

[19] T. J. Gluckman , N. M. Bhave , L. A. Allen , et al., “2022 ACC Expert Consensus Decision Pathway on Cardiovascular Sequelae of COVID‐19 in Adults: Myocarditis and Other Myocardial Involvement, Post‐Acute Sequelae of SARS‐CoV‐2 Infection, and Return to Play: A Report of the American College of Cardiology Solution Set Oversight Committee,” Journal of the American College of Cardiology 79, no. 17 (2022): 1717–1756. 35307156 10.1016/j.jacc.2022.02.003 PMC8926109

[20] M. J. Page , J. E. McKenzie , P. M. Bossuyt , et al., “The PRISMA 2020 Statement: An Updated Guideline for Reporting Systematic Reviews,” BMJ 372 (2021): n71. 33782057 10.1136/bmj.n71 PMC8005924

[21] Institute JB , “Checklist for Cohort Studies 2020,” (2020), https://jbi.global/critical‐appraisal‐tools.

[22] I. C. H. Lam , C. K. H. Wong , R. Zhang , et al., “Long‐Term Post‐Acute Sequelae of COVID‐19 Infection: A Retrospective, Multi‐Database Cohort Study in Hong Kong and the UK,” EClinicalMedicine 60 (2023): 1–10. 10.1016/j.eclinm.2023.102000 PMC10173760 37197226

[23] L. Ojeda‐Fernández , M. Baviera , A. Foresta , et al., “Impact of First and Second/Third Wave of COVID‐19 Pandemic on Post‐Acute Cardiovascular Outcomes in Lombardy,” Frontiers in Cardiovascular Medicine 10 (2023): 1244002. 37781303 10.3389/fcvm.2023.1244002 PMC10536134

[24] S. E. Daugherty , Y. Guo , K. Heath , et al., “Risk of Clinical Sequelae After the Acute Phase of SARS‐CoV‐2 Infection: Retrospective Cohort Study,” BMJ 373 (2021): n1098. 34011492 10.1136/bmj.n1098 PMC8132065

[25] S. Mabila , D. Patel , M. Fan , et al., “Post‐Acute Sequalae of COVID‐19 and Cardiac Outcomes in US Military Members,” International Journal of Cardiology Cardiovascular Risk and Prevention 17 (2023): 200183. 36936859 10.1016/j.ijcrp.2023.200183 PMC10014478

[26] W. Wang , C.‐Y. Wang , S.‐I. Wang , and J. C.‐C. Wei , “Long‐Term Cardiovascular Outcomes in COVID‐19 Survivors Among Non‐Vaccinated Population: A Retrospective Cohort Study From the TriNetX US Collaborative Networks,” EClinicalMedicine 53 (2022): 1–11. 10.1016/j.eclinm.2022.101619 PMC9366236 35971425

[27] Y. Xie , E. Xu , B. Bowe , and Z. Al‐Aly , “Long‐Term Cardiovascular Outcomes of COVID‐19,” Nature Medicine 28, no. 3 (2022): 583–590. 10.1038/s41591-022-01689-3 PMC8938267 35132265

[28] E. Rezel‐Potts , A. Douiri , X. Sun , P. J. Chowienczyk , A. M. Shah , and M. C. Gulliford , “Cardiometabolic Outcomes up to 12 Months After COVID‐19 Infection. A Matched Cohort Study in the UK,” PLoS Medicine 19, no. 7 (2022): e1004052. 35853019 10.1371/journal.pmed.1004052 PMC9295991

[29] E. Y. F. Wan , S. Mathur , R. Zhang , et al., “Association of COVID‐19 With Short‐ and Long‐Term Risk of Cardiovascular Disease and Mortality: A Prospective Cohort in UK Biobank,” Cardiovascular Research 119, no. 8 (2023): 1718–1727. 36652991 10.1093/cvr/cvac195

[30] J. T. Lim , W. Liang En , A. T. Tay , et al., “Long‐Term Cardiovascular, Cerebrovascular, and Other Thrombotic Complications in COVID‐19 Survivors: A Retrospective Cohort Study,” Clinical Infectious Diseases 78, no. 1 (2024): 70–79. 37746872 10.1093/cid/ciad469 PMC10810710

[31] L. E. Wee , J. T. Lim , A. T. Tay , et al., “Long‐Term Cardiovascular, Cerebrovascular, and Thrombotic Complications After SARS‐CoV‐2‐Omicron Infection: A Retrospective Cohort Study,” Clinical Microbiology and Infection 30, no. 10 (2024): 1319–1326. 38908748 10.1016/j.cmi.2024.06.011

[32] L. Ortega‐Paz , V. Arévalos , D. Fernández‐Rodríguez , et al., “One‐Year Cardiovascular Outcomes After Coronavirus Disease 2019: The Cardiovascular COVID‐19 Registry,” PLoS One 17, no. 12 (2022): e0279333. 36583998 10.1371/journal.pone.0279333 PMC9803130

[33] C. Tintore , J. Cuartero , A. Camps‐Vilaró , et al., “Increased Risk of Arrhythmias, Heart Failure, and Thrombosis in SARS‐CoV‐2 Positive Individuals Persists at One Year Post‐Infection,” Computational and Structural Biotechnology Journal 24 (2024): 476. 39050244 10.1016/j.csbj.2024.06.024 PMC11266869

[34] Organization WH , International Statistical Classification of Diseases and Related Health Problems, 11th ed. Geneva: World Health Organization (2024).

[35] G. F. Romiti , B. Corica , G. Y. Lip , and M. Proietti , “Prevalence and Impact of Atrial Fibrillation in Hospitalized Patients With COVID‐19: A Systematic Review and Meta‐Analysis,” Journal of Clinical Medicine 10, no. 11 (2021): 2490. 34199857 10.3390/jcm10112490 PMC8200114

[36] M. Zuin , L. Ojeda‐Fernández , G. Torrigiani , and M. Bertini , “Risk of Incident Atrial Fibrillation After COVID‐19 Infection: A Systematic Review and Meta‐Analysis,” Heart Rhythm 21 (2024): 1613–1620. 38636931 10.1016/j.hrthm.2024.04.064

[37] B. Rosh , I. Naoum , O. Barnett‐Griness , R. Najjar‐Debbiny , and W. Saliba , “Association Between SARS‐CoV‐2 Infection and New‐Onset Atrial Fibrillation,” International Journal of Cardiology 392 (2023): 131298. 37652274 10.1016/j.ijcard.2023.131298

[38] J. Aranyó , V. Bazan , G. Lladós , et al., “Inappropriate Sinus Tachycardia in Post‐COVID‐19 Syndrome,” Scientific Reports 12, no. 1 (2022): 298. 34996973 10.1038/s41598-021-03831-6 PMC8741896

[39] J. Arano Llach , V. Victor Bazan , G. Gemma Llados , et al., “Inappropriate Sinus Tachycardia in Post‐Covid‐19 Syndrome,” EP Europace 23 (2021): 23. 10.1038/s41598-021-03831-6 PMC8741896 34996973

[40] C. Umeh , C. Giberson , S. Kumar , M. Aseri , and P. Barve , “A Multicenter Retrospective Analysis on the Etiology of Bradycardia in COVID‐19 Patients,” Cureus 14, no. 1 (2022): 1–8. 10.7759/cureus.21294 PMC8846448 35186556

[41] F. Stancampiano , M. Omer , D. Harris , et al., “Clinical Characteristics and Outcomes of Patients Hospitalized for COVID‐19 Pneumonia Who Developed Bradycardia,” Southern Medical Journal 114, no. 7 (2021): 432–437. 34215897 10.14423/SMJ.0000000000001269 PMC8231014

[42] M. Zhou , C.‐K. Wong , K.‐C. Un , et al., “Cardiovascular Sequalae in Uncomplicated COVID‐19 Survivors,” PLoS One 16, no. 2 (2021): e0246732. 33571321 10.1371/journal.pone.0246732 PMC7877588

[43] F. Afroze , S. M. Arafat , C. M. Ahmed , et al., “Features and Risk Factors of Post‐COVID‐19 Syndrome: Findings From a Longitudinal Study in Bangladesh,” Lancet Regional Health‐Southeast Asia 11 (2023): 100134. 36575774 10.1016/j.lansea.2022.100134 PMC9780633

[44] R. Gopinathannair , B. Olshansky , M. K. Chung , et al., “Cardiac Arrhythmias and Autonomic Dysfunction Associated With COVID‐19: A Scientific Statement From the American Heart Association,” Circulation 150, no. 21 (2024): e449–e465. 39397661 10.1161/CIR.0000000000001290 PMC11734731

[45] H.‐W. Suh , C.‐Y. Kwon , and B. Lee , “Long‐Term Impact of COVID‐19 on Heart Rate Variability: A Systematic Review of Observational Studies,” Health 11, no. 8 (2023): 1095. 10.3390/healthcare11081095 PMC10137929 37107929

[46] Z. Tan , S. Huang , K. Mei , et al., “The Prevalence and Associated Death of Ventricular Arrhythmia and Sudden Cardiac Death in Hospitalized Patients With COVID‐19: A Systematic Review and Meta‐Analysis,” Frontiers in Cardiovascular Medicine 8 (2022): 8. 10.3389/fcvm.2021.795750 PMC8814312 35127861

[47] C. B. Ingul , J. Grimsmo , A. Mecinaj , et al., “Cardiac Dysfunction and Arrhythmias 3 Months After Hospitalization for COVID‐19,” Journal of the American Heart Association 11, no. 3 (2022): e023473. 35048715 10.1161/JAHA.121.023473 PMC9238505

[48] C. I. Rosca , H. S. Branea , A. Sharma , et al., “Rhythm Disturbances in Post‐Acute COVID‐19 Syndrome in Young Men Without Pre‐Existing Known Cardiovascular Disease—A Case Series,” Biomedicine 11, no. 4 (2023): 1146. 10.3390/biomedicines11041146 PMC10136152 37189764

[49] A. H. El Hajjar , M. C. El Helou , A. Bayat , et al., “Ventricular Tachycardia as a Late Complication of COVID‐19 in a Young Patient With No History of Cardiovascular Disease,” CJC Open 6, no. 5 (2024): 721–724. 38846438 10.1016/j.cjco.2024.01.010 PMC11150940

[50] R. M. Hamdy , M. Samy , and H. S. Mohamed , “Clinical Utility of Ambulatory ECG Monitoring and 2D‐Ventricular Strain for Evaluation of Post‐COVID‐19 Ventricular Arrhythmia,” BMC Cardiovascular Disorders 24, no. 1 (2024): 429. 39148011 10.1186/s12872-024-03982-0 PMC11328462

[51] M. Augustin , P. Schommers , M. Stecher , et al., “Post‐COVID Syndrome in Non‐Hospitalised Patients With COVID‐19: A Longitudinal Prospective Cohort Study,” Lancet Regional Health 6 (2021): 1–8. 10.1016/j.lanepe.2021.100122 PMC8129613 34027514

[52] C. Fernández‐de‐las‐Peñas , D. Palacios‐Ceña , V. Gómez‐Mayordomo , et al., “Prevalence of Post‐COVID‐19 Symptoms in Hospitalized and Non‐Hospitalized COVID‐19 Survivors: A Systematic Review and Meta‐Analysis,” European Journal of Internal Medicine 92 (2021): 55–70. 34167876 10.1016/j.ejim.2021.06.009 PMC8206636

[53] C. Fernández‐de‐las‐Peñas , J. Rodríguez‐Jiménez , I. Cancela‐Cilleruelo , et al., “Post–COVID‐19 Symptoms 2 Years After SARS‐CoV‐2 Infection Among Hospitalized vs Nonhospitalized Patients,” JAMA Network Open 5, no. 11 (2022): e2242106. 36378309 10.1001/jamanetworkopen.2022.42106 PMC9667330

[54] M. Peghin , A. Palese , M. Venturini , et al., “Post‐COVID‐19 Symptoms 6 Months After Acute Infection Among Hospitalized and Non‐Hospitalized Patients,” Clinical Microbiology and Infection 27, no. 10 (2021): 1507–1513. 34111579 10.1016/j.cmi.2021.05.033 PMC8180450

[55] T. L. Wiemken , L. J. McGrath , K. M. Andersen , et al., “Coronavirus Disease 2019 Severity and Risk of Subsequent Cardiovascular Events,” Clinical Infectious Diseases 76, no. 3 (2023): e42–e50. 35984816 10.1093/cid/ciac661 PMC9907540

