# Supplementary material for: Increased Arrhythmia Risk in Long COVID: A Systematic Review and Meta‐Analysis
Source: J Arrhythm. 2026 Feb 9;42(1):e70278. doi: 10.1002/joa3.70278 (PMC12887432; doi:10.1002/joa3.70278)
Supplement: Supplementary file 1 — Data S1: joa370278‐sup‐0001‐supinfo.docx. [file JOA3-42-e70278-s001.docx]

**Supplementary Material**

**Increased Arrhythmia Risk in Long COVID: A Systematic Review and Meta-Analysis**

**Table of Contents**

[Supplementary Table 1. Search Strategy of the Systematic Review 2](#_Toc214500182)

[Supplementary Table 2. Quality Assessment of the included studies in the systematic review 3](#_Toc214500183)

[Supplementary Figure 1. Funnel plot for the risk of overall arrhythmias in long COVID 4](#_Toc214500184)

[Supplementary Figure 2. Sensitivity analysis for the risk of overall arrhythmias in long COVID 5](#_Toc214500185)

[Supplementary Figure 3. Funnel plot for the risk of atrial fibrillation in long COVID 6](#_Toc214500186)

[Supplementary Figure 4. Sensitivity analysis for the risk of atrial fibrillation in long COVID 7](#_Toc214500187)

[Supplementary Figure 5. Funnel plot for the risk of sinus tachycardia in long COVID 8](#_Toc214500188)

[Supplementary Figure 6. Sensitivity analysis for the risk of sinus tachycardia in long COVID 9](#_Toc214500189)

[Supplementary Figure 7. Funnel plot for the risk of sinus bradycardia in long COVID 10](#_Toc214500190)

[Supplementary Figure 8. Sensitivity analysis for the risk of sinus bradycardia in long COVID 11](#_Toc214500191)

[References 12](#_Toc214500192)

| **Search query** | | **Results (August 24th, 2025)** |
| --- | --- | --- |
| **PubMed** | | |
| #1 | (arrhythmia[tiab] OR tachycardia[tiab] OR "Arrhythmias, Cardiac"[Mesh] OR "Tachycardia"[Mesh]) | 304,550 |
| #2 | ("Post-Acute COVID-19 Syndrome"[Mesh] OR "Post-Acute COVID-19 Syndrome"[tiab] OR "long covid"[tiab] OR "long haul covid"[tiab] OR "long hauler covid"[tiab] OR "post-acute sequelae of covid"[tiab]) | 9,225 |
| #3 | #1 AND #2 | ***239*** |
| **SCOPUS** | | |
| #1 | ALL (arrhythmia) OR ALL (tachycardia) | 662,242 |
| #2 | ALL("COVID-19 Syndrome, Post-Acute") OR ALL("Post-Acute COVID-19 Syndromes") OR ALL("Long Haul COVID-19") OR ALL("COVID-19, Long Haul") OR ALL("Long Haul COVID 19") OR ALL("Long Haul COVID-19s") OR ALL("Post Acute COVID-19 Syndrome") OR ALL("Post Acute COVID 19 Syndrome") OR ALL("Long COVID") OR ALL("Post-Acute Sequelae of SARS-CoV-2 Infection") OR ALL("Post Acute Sequelae of SARS CoV 2 Infection") OR ALL("Post-COVID Conditions") OR ALL("Post COVID Conditions") OR ALL("Post-COVID Condition") OR ALL("Long-Haul COVID") OR ALL("COVID, Long-Haul") OR ALL("Long Haul COVID") OR ALL("Long-Haul COVIDs") | 32,430 |
| #3 | #1 AND #2 | ***2,551 + 106 preprints*** |
| **Web of Science** | | |
| #1 | (ALL=(arrhythmia)) OR ALL=(tachycardia) | 204,688 |
| #2 | (((((((((((((((((ALL=("COVID-19 Syndrome, Post-Acute")) OR ALL=("Post-Acute COVID-19 Syndromes")) OR ALL=("Long Haul COVID-19")) OR ALL=("COVID-19, Long Haul")) OR ALL=("Long Haul COVID 19")) OR ALL=("Long Haul COVID-19s")) OR ALL=("Post Acute COVID-19 Syndrome")) OR ALL=("Post Acute COVID 19 Syndrome")) OR ALL=("Long COVID")) OR ALL=("Post-Acute Sequelae of SARS-CoV-2 Infection")) OR ALL=("Post Acute Sequelae of SARS CoV 2 Infection")) OR ALL=("Post-COVID Conditions")) OR ALL=("Post COVID Conditions")) OR ALL=("Post-COVID Condition")) OR ALL=("Long-Haul COVID")) OR ALL=("COVID, Long-Haul")) OR ALL=("Long Haul COVID")) OR ALL=("Long-Haul COVIDs") | 9,538 |
| #3 | #1 AND #2 | ***288*** |
| **Science Direct** | | |
| #1 | (Arrhythmia OR tachycardia) | 420,918 |
| #2 | ("Post-Acute COVID-19 Syndrome" OR "long covid" OR "long haul covid" OR "long hauler covid" OR "post-acute sequelae of covid") | 6,876 |
| #3 | #1 AND #2 | ***960*** |
| **TOTAL** | | ***4038 + 106*** |
| **DUPLICATES** | | ***1113*** |
| **TOTAL AFTER REMOVING DUPLICATES** | | ***3031*** |

# Supplementary Table 1. Search Strategy of the Systematic Review

|  | **Question** | **Were the two groups similar and recruited from the same population?** | **Were the exposures measured similarly to assign people to exposed and unexposed groups?** | **Was the exposure measured validly and reliably?** | **Were confounding factors identified?** | **Were strategies to deal with confounding factors stated?** | **Were the groups/participants free of the outcome at the start of the study (or at the moment of exposure)?** | **Were the outcomes measured validly and reliably?** | **Was the follow-up time reported sufficient to be long enough for outcomes to occur?** | **Was follow-up complete, and if not, were the reasons for loss to follow-up described and explored?** | **Were strategies to address incomplete follow-up utilized?** | **Was appropriate statistical analysis used?** |  |
| --- | --- | --- | --- | --- | --- | --- | --- | --- | --- | --- | --- | --- | --- |
|  | **Author** | 1 | 2 | 3 | 4 | 5 | 6 | 7 | 8 | 9 | 10 | 11 | TOTAL |
| **STUDIES** | Xie Y. (1) | yes | yes | yes | yes | unclear | yes | yes | yes | yes | yes | yes | 10 |
|  | Wang W. (2) | yes | yes | yes | yes | yes | yes | yes | yes | yes | yes | yes | 11 |
|  | Ortega-Paz L. (3) | yes | yes | yes | yes | yes | yes | yes | yes | yes | yes | yes | 11 |
|  | Mabila S. (4) | yes | yes | yes | yes | yes | yes | yes | yes | yes | yes | yes | 11 |
|  | Wan E. (5) | yes | yes | yes | yes | yes | yes | yes | yes | yes | yes | yes | 11 |
|  | Lim J. (6) | yes | yes | yes | yes | yes | yes | yes | yes | yes | yes | yes | 11 |
|  | Tintore C. (7) | yes | yes | yes | yes | yes | yes | yes | yes | yes | yes | yes | 11 |
|  | Lam I. (8) | yes | yes | yes | yes | yes | yes | yes | yes | yes | yes | yes | 11 |
|  | Ojeda-Fernandez L. (9) | yes | yes | yes | yes | yes | yes | yes | yes | yes | yes | yes | 11 |
|  | Wee L. (10) | yes | yes | yes | yes | yes | yes | yes | yes | yes | yes | yes | 11 |
|  | Daugherty S. (11) | yes | yes | yes | yes | yes | yes | yes | yes | yes | yes | yes | 11 |
|  | Rezel-Potts E. (12) | yes | yes | yes | yes | yes | yes | yes | yes | yes | yes | yes | 11 |

# Supplementary Table 2. Quality Assessment of the included studies in the systematic review


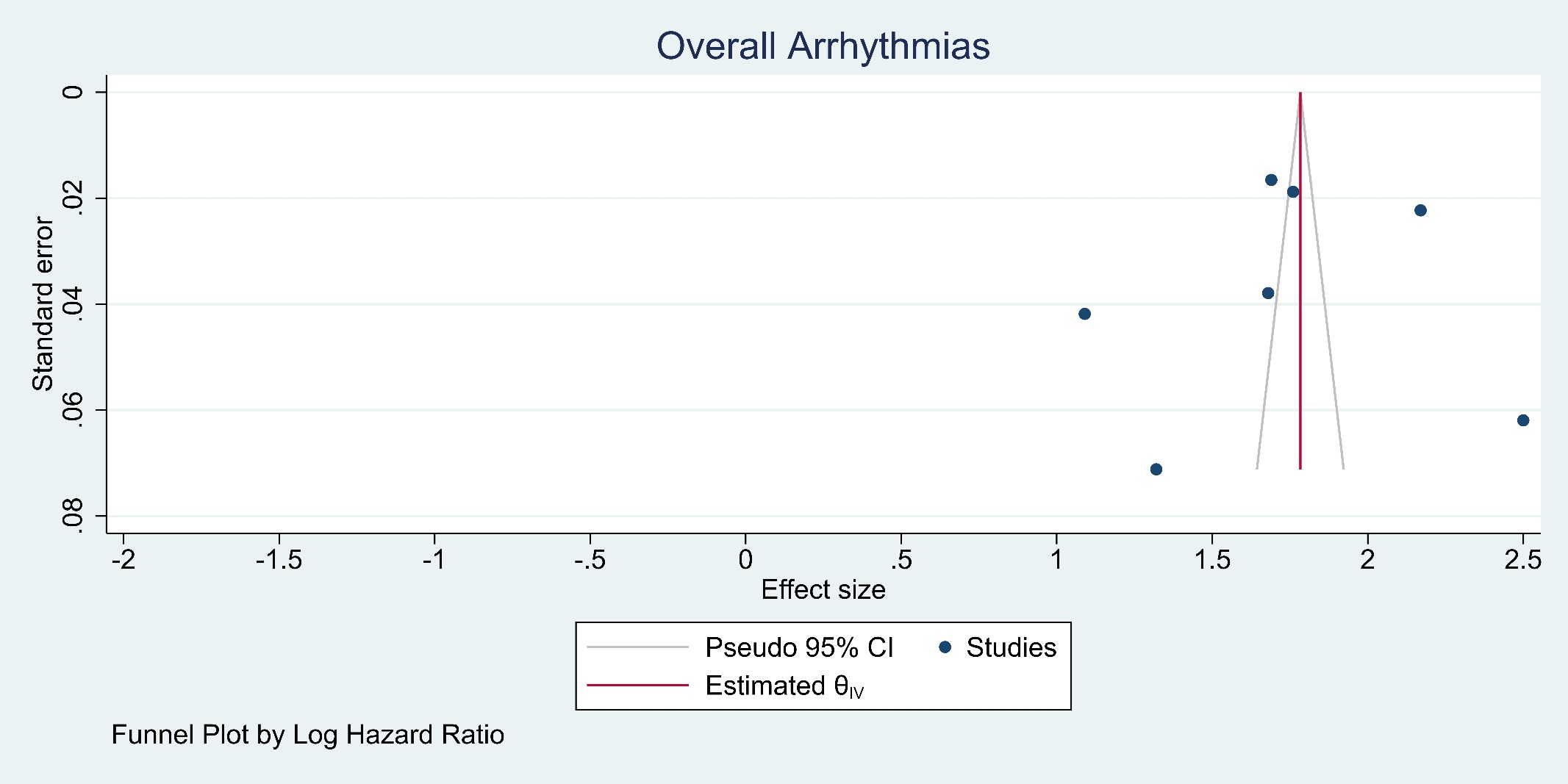


# Supplementary Figure 1. Funnel plot for the risk of overall arrhythmias in long COVID


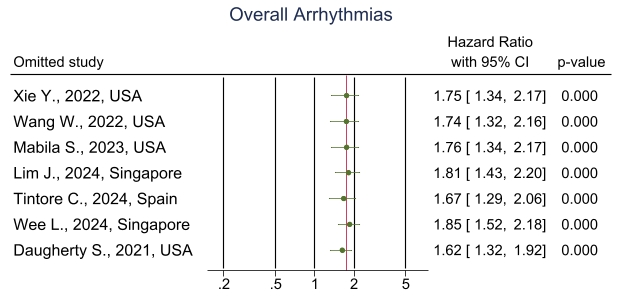


# Supplementary Figure 2. Sensitivity analysis for the risk of overall arrhythmias in long COVID


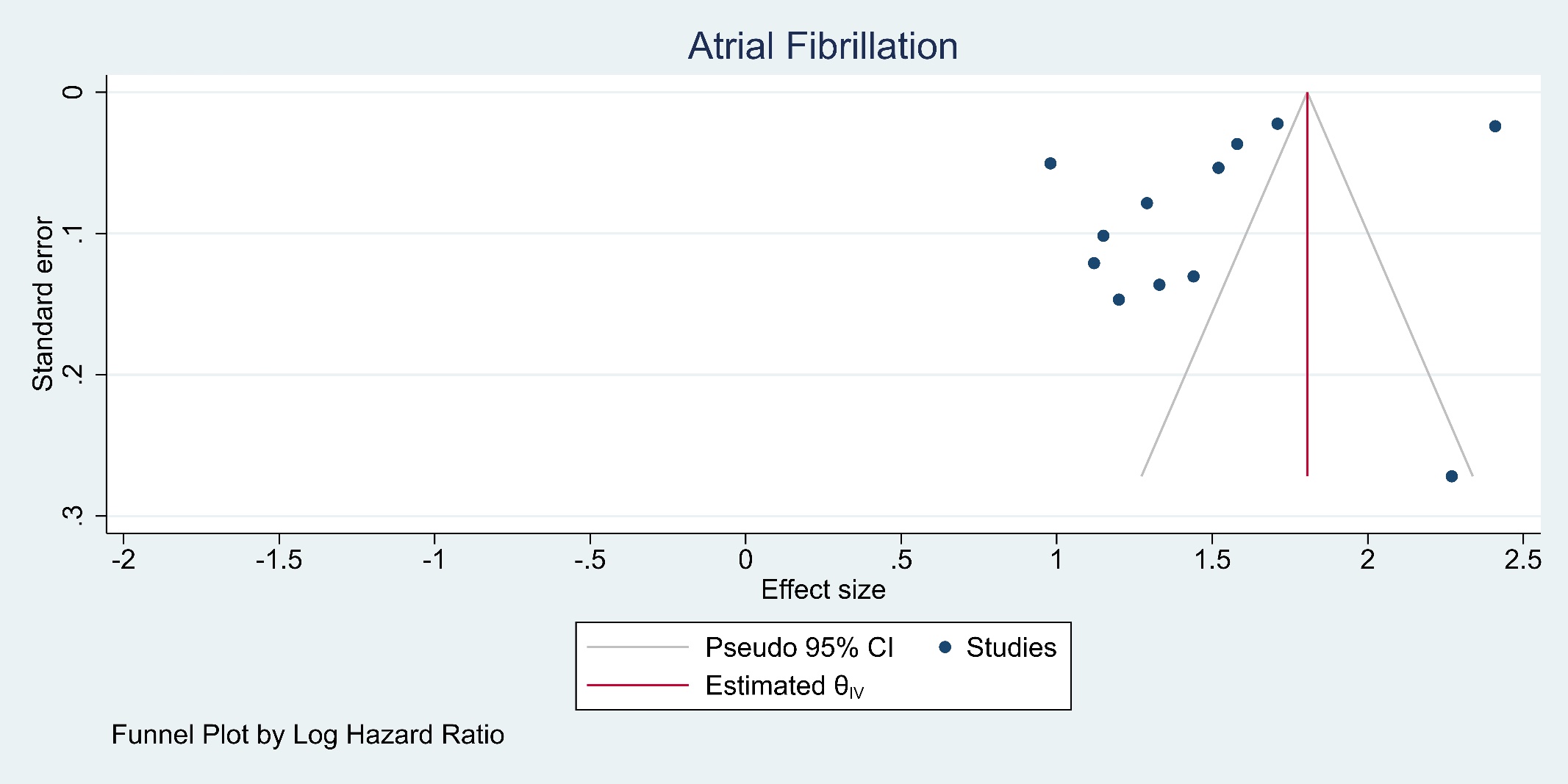


# Supplementary Figure 3. Funnel plot for the risk of atrial fibrillation in long COVID


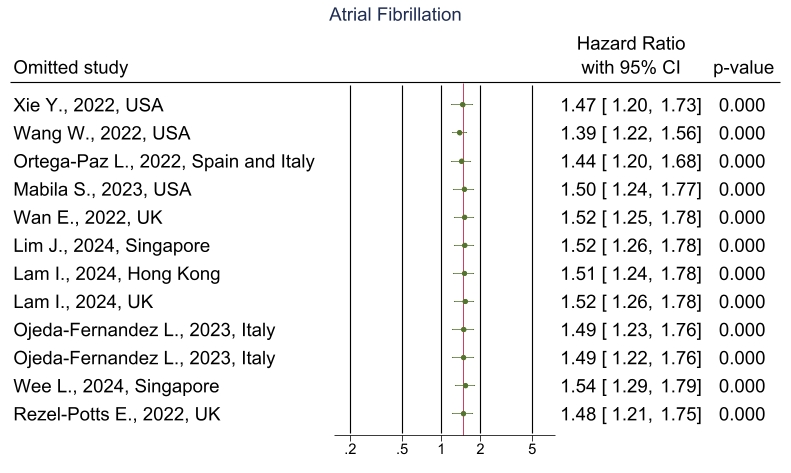
**Supplementary Figure 4.** Sensitivity analysis for the risk of atrial fibrillation in long COVID


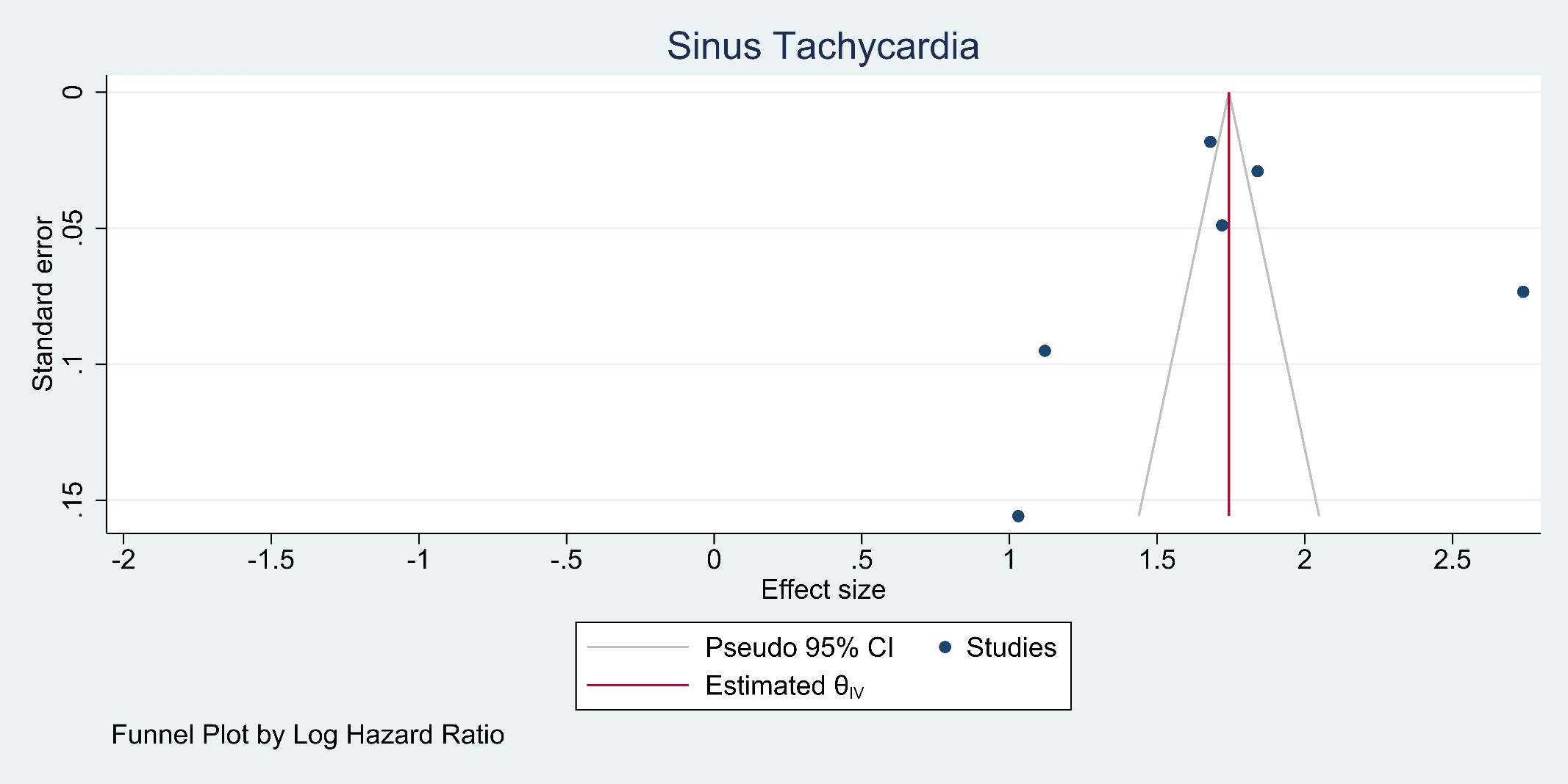


# Supplementary Figure 5. Funnel plot for the risk of sinus tachycardia in long COVID


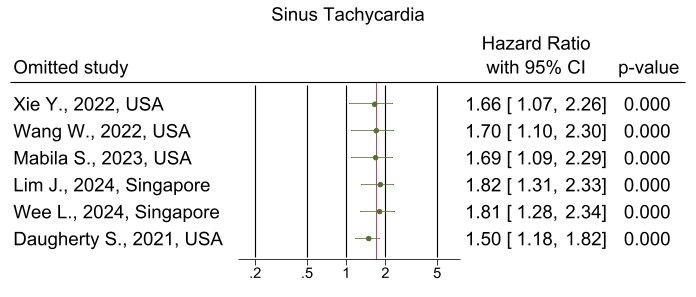


# Supplementary Figure 6. Sensitivity analysis for the risk of sinus tachycardia in long COVID


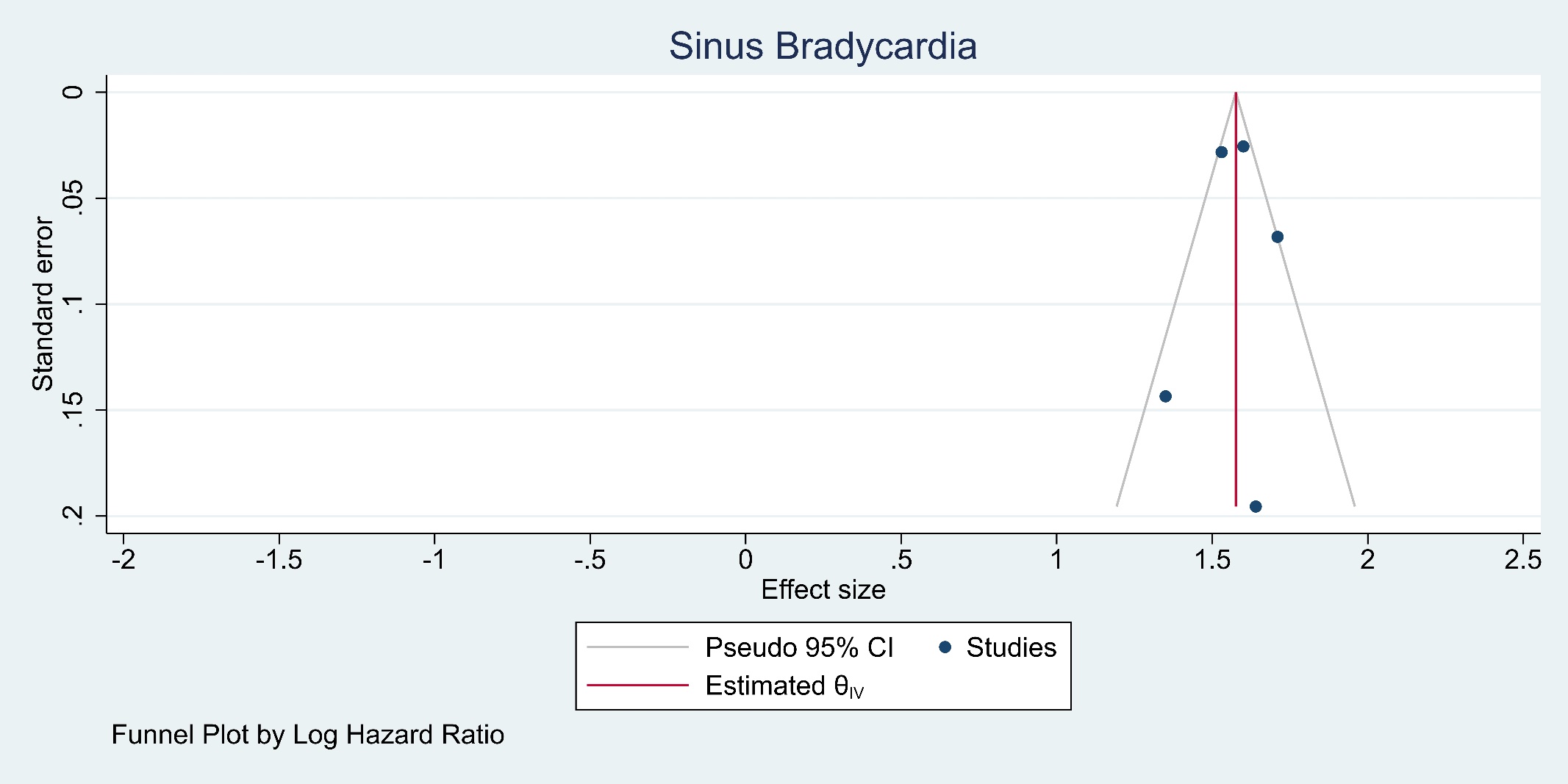


# Supplementary Figure 7. Funnel plot for the risk of sinus bradycardia in long COVID


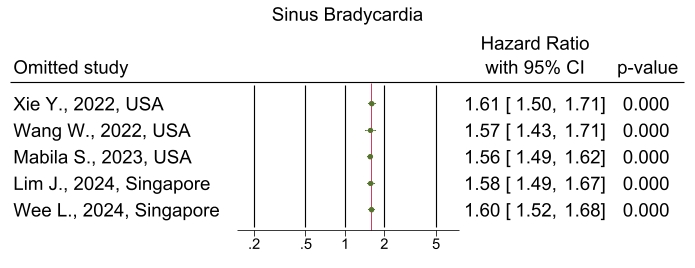


# Supplementary Figure 8. Sensitivity analysis for the risk of sinus bradycardia in long COVID

# References:

1. Xie Y, Xu E, Bowe B, Al-Aly Z. Long-term cardiovascular outcomes of COVID-19. Nature medicine. 2022;28(3):583-90.

2. Wang W, Wang C-Y, Wang S-I, Wei JC-C. Long-term cardiovascular outcomes in COVID-19 survivors among non-vaccinated population: a retrospective cohort study from the TriNetX US collaborative networks. EClinicalMedicine. 2022;53.

3. Ortega-Paz L, Arévalos V, Fernández-Rodríguez D, Jiménez-Díaz V, Bañeras J, Campo G, et al. One-year cardiovascular outcomes after coronavirus disease 2019: The cardiovascular COVID-19 registry. PLoS One. 2022;17(12):e0279333.

4. Mabila S, Patel D, Fan M, Stahlman S, Seliga N, Nowak G, et al. Post–acute sequalae of COVID-19 and cardiac outcomes in US military members. International Journal of Cardiology Cardiovascular Risk and Prevention. 2023;17:200183.

5. Wan EYF, Mathur S, Zhang R, Yan VKC, Lai FTT, Chui CSL, et al. Association of COVID-19 with short-and long-term risk of cardiovascular disease and mortality: a prospective cohort in UK Biobank. Cardiovascular research. 2023;119(8):1718-27.

6. Lim JT, Liang En W, Tay AT, Pang D, Chiew CJ, Ong B, et al. Long-term cardiovascular, cerebrovascular, and other thrombotic complications in COVID-19 survivors: a retrospective cohort study. Clinical Infectious Diseases. 2024;78(1):70-9.

7. Tintore C, Cuartero J, Camps-Vilaró A, Subirana I, Elosua R, Marrugat J, et al. Increased risk of arrhythmias, heart failure, and thrombosis in SARS-CoV-2 positive individuals persists at one year post-infection. Computational and Structural Biotechnology Journal. 2024.

8. Lam ICH, Wong CKH, Zhang R, Chui CSL, Lai FTT, Li X, et al. Long-term post-acute sequelae of COVID-19 infection: a retrospective, multi-database cohort study in Hong Kong and the UK. EClinicalMedicine. 2023;60.

9. Ojeda-Fernández L, Baviera M, Foresta A, Tettamanti M, Zambon A, Macaluso G, et al. Impact of first and second/third wave of COVID-19 pandemic on post-acute cardiovascular outcomes in Lombardy. Frontiers in Cardiovascular Medicine. 2023;10:1244002.

10. Wee LE, Lim JT, Tay AT, Pang D, Chiew CJ, Chia YW, et al. Long-term cardiovascular, cerebrovascular, and thrombotic complications after SARS-CoV-2-Omicron infection: a retrospective cohort study. Clinical Microbiology and Infection. 2024;30(10):1319-26.

11. Daugherty SE, Guo Y, Heath K, Dasmariñas MC, Jubilo KG, Samranvedhya J, et al. Risk of clinical sequelae after the acute phase of SARS-CoV-2 infection: retrospective cohort study. Bmj. 2021;373:n1098.

12. Rezel-Potts E, Douiri A, Sun X, Chowienczyk PJ, Shah AM, Gulliford MC. Cardiometabolic outcomes up to 12 months after COVID-19 infection. A matched cohort study in the UK. PLoS Med. 2022;19(7):e1004052.
